# The mid-term outcome of primary open valvotomy for critical aortic stenosis in early infancy - a retrospective single center study over 18 years

**DOI:** 10.1186/s13019-016-0509-9

**Published:** 2016-08-02

**Authors:** Claire Galoin-Bertail, André Capderou, Emre Belli, Lucile Houyel

**Affiliations:** 1 Centre de Référence Malformations Cardiaques Congénitales Complexes-M3C, Centre Chirurgical Marie-Lannelongue, INSERM U 999, Université Paris-Sud, 133 Avenue Résistance, 92350 Le Plessis Robinson, E.U. France; 2 Institut Hospitalier Jacques Cartier, 6 Avenue du Loyer Lambert, 91300 Massy, E.U. France

## Abstract

**Background:**

The objective of this study was to examine early and long-term results of surgical aortic valvotomy in neonates and infants aged less than four months and to identify predictors of outcome.

**Methods:**

Between August 1994 and April 2012, 83 consecutive patients younger than 4 months of age underwent open heart valvotomy for critical aortic stenosis in our institution. Median age was 17 days (range 0-111 days). We examined clinical records to establish determinants of outcome and illustrate long-term results.

**Results:**

Fifty-six patients (67 %) were neonates. Associated cardiac malformations were found in 24 patients (29 %), including multilevel left heart obstruction in 5. The median follow-up was 4.2 years. The time-related survival rate was 87 and 85 % at 5 and 15 years, respectively. The time-related survival without reintervention was respectively 51, 35 and 18 % at 5, 10 and 15 years. The time-related survival without aortic valve replacement was respectively 67, 54 and 39 % at 5, 10 and 15 years. Ventricular dysfunction (*p* = 0.04), delayed sternal closure (*p* = 0.007), endocardial fibroelastosis (*p* = 0.02) and low z-score of the aortic annulus (*p* = 0.04) were found predictors of global mortality. Ventricular dysfunction (*p* = 0.01) and endocardial fibroelastosis (*p* = 0.04) were found predictors of reintervention.

**Conclusions:**

The experience, in our center, on the management of critical aortic stenosis, shows a low early and late mortality, but the aortic valvotomy is a palliative procedure and we see unfortunately a high rate of reintervention among which the aortic valve replacement. These results suggest to reconsider the use of aortic balloon valvotomy, and particularly for the neonates with a low cardiac output in order to avoid the myocardial stress and the neurological injury due to the cardiopulmonary bypass.

## Background

Management of critical aortic stenosis in infants is still a controversial issue [1, 2]. When the patient is considered suitable for a biventricular repair, several options are available: conservative surgery including open heart valvotomy or, in some centers, closed transventricular aortic valvotomy [3]; or transcatheter balloon dilation.

During surgical open heart valvotomy, the surgeon has direct access to the morphology of the aortic valve and can adapt surgical strategy to individual valvular anatomy. In addition to dividing the zones of commissural fusion, thick and dysplastic leaflets can be thinned, improving valvular mobility. After surgery there is generally a low risk of aortic regurgitation with moderate residual gradient [4].

In contrast, transcatheter balloon dilation does not permit direct vision of the valve and tears aortic leaflets in a random way, usually at the weakest part of the valve and not necessarily at the level of the fused commissures. Despite its “blind” nature, transcatheter balloon dilation is now favored by most teams in the neonate and young infant as a first-choice procedure, because of its greater facility to carry out. After percutaneous balloon valvuloplasty, there is a higher risk of significant aortic regurgitation but generally a lower risk of residual stenosis. Progress in catheter procedures using new generation balloons of different sizes more adapted to the individual anatomic situation of the infant makes the difference between surgical commissurotomy and balloon valvuloplasty not evident [5–8].

Valvotomy of any kind is a palliative procedure, and reinterventions remain frequent [9].

In our institution, the approach to the neonate and young infant with critical aortic stenosis eligible for biventricular repair (size of aortic annulus not less than five millimeters) was almost exclusively open surgical valvotomy since 1994. Some percutaneous balloon valvuloplasties were performed from 1986 to 1993 and abandoned because of occurrence of acute aortic insufficiency.

The purpose of this study was to evaluate the results of this deliberate surgical approach, by analyzing the mid-term outcome after surgical valvotomy in young infants less than four months of age. In the current area of the percutaneous dilation of the aortic valve, we would like to know if we made the best choice of management of critical aortic stenosis.

## Methods

Between August 1994 and April 2012, 83 consecutive patients younger than 4 months of age (19 girls, 64 boys) underwent open heart valvotomy for critical aortic stenosis in our institution. Median age was 17 days (range 0–111 days). Fifty-six patients (67 %) were neonates. Median weight at operation was 3.5 kg (range 1.8–6.9 kg).

### Definition of study group

Critical aortic stenosis was an isolated lesion in 59 patients (71 %). Associated cardiac malformations were found in 24 patients (29 %), including multilevel left heart obstruction in 5, aortic coarctation in 8, atrial septal defect in 5, interruption of the aortic arch in 1 (IAA), ventricular septal defect in 2, mitral stenosis in 2, a mild form of hypoplastic left heart syndrome (HLHS) in 1, and supra valvular aortic stenosis with associated stenosis of the right pulmonary artery in 1.

The surgical threshold of intervention is a mean gradient ≥ 50 mmHg or less, if the patient presents a severe left ventricular dysfunction and/or an associated cardiac malformation requiring a concomitant surgery.

The neonate and young infant with critical aortic stenosis were eligible for biventricular repair if the size of the aortic annulus didn't measure less than five millimeters.

### Clinical features

Nine patients were directly admitted in the intensive care unit because of cardiorespiratory failure. Eight needed ventilation support and nine needed inotropic support. Ductal dependency was present in 14 patients who required prostaglandin E1 treatment.

The malformation had been diagnosed prenatally in 14 patients (17 %).

The pressure gradient across the aortic valve was measured by transthoracic echocardiography coupled with continuous wave Doppler technique. Preoperative peak and mean aortic valve gradient were respectively 86 mmHg (range 20 to 190 mmHg) and 53 mmHg (range 14 to 106 mmHg). Aortic regurgitation grade ¼, assessed by color-flow Doppler, was present in two patients (2,4 %). Four patients had associated subaortic stenosis (4.8 %). Three of four patients with subaortic stenosis were included in the associated cardiac malformations described in definition of study group section (one aortic coarctation and two multilevel left heart obstructions) except one who has an isolated aortic stenosis.

The median z-score of the aortic annulus was −1.9 (range −7.5 to 1.6), the median z-score of the ascending aorta was 2.1 (range −3.8 to 4.7), the median z-score of left ventricle end-diastolic diameter (LVEDD) was 0.5 (range −3.9 to 7.2) [10].

Caution should be used with these measures because, on one hand, it's an historic series with a downstream calculation of z-score, and, on the other hand, according to the method of calculation of the z-score, the value of the latter can change a lot.

Thirty-seven patients had left ventricular dysfunction (45 %), defined as a shortening fraction (SF) < 30 %. The median SF was 35 % (range 8 to 61 %). Endocardial fibroelastosis (EFE) was present in 31 % of patients (determined by echocardiography and confirmed by the surgeon; The extend of EFE was not detailed).

### Morphology of the aortic valve assessed at surgery

The aortic valve was bicuspid in 70 patients (84 %), monocuspid in seven patients (9 %) and tricuspid in six patients (7 %).

### Surgical technique

After median sternotomy, right atrial cannulation, and aortic cannulation, all patients were placed on normothermic cardiopulmonary bypass (CPB). Myocardial protection was obtained with hyper potassic antegrade blood repeated at ten minutes intervals; Through a transverse aortotomy, a careful commissurotomy of the aortic valve was performed in order to avoid appearance of aortic regurgitation (AR). Obstructive myxomatous and fibrous nodules were removed from the leaflets. Median CPB time was 42 min (range 13–260 min), median aortic cross-clamping time was 20 min (range 6–69 min).

Concomitant surgery (18 %) included aortic coarctation repair in 11 patients, IAA repair in one patient, closure of ventricular septal defect in 1, closure of atrial septal defect in 3, resection of supra-mitral membrane in 1, mitral commissurotomy in 1 and pulmonary artery banding in 1.

The surgeon had to enlarge the sinotubular junction with a patch in 17 patients (20.5 %) without supra-aortic stenosis described pre-operatively. The decision was taken by the surgeons in view of the observations during open heart valvotomy.

Delayed sternal closure was required in 16 patients (19 %).

### Follow-up

Hospital survivors were examined in outpatient clinics at regular intervals by their pediatric cardiologist. Two-dimensional echocardiography and Doppler studies were routinely performed before discharge from the hospital and during each outpatient visit. The transvalvular aortic peak pressure gradient was calculated using the simplified Bernoulli equation. Color-flow Doppler imaging was used to analyze the degree of AR, using jet width and end-diastolic velocity of the retrograde flow in the descending aorta. The median follow-up was 4.2 years (range 1 day to 17.7 years). Five patients were lost of follow-up. Early death was defined as death in the hospital or within 30 days after the operation.

### Statistical analysis

The data were imported into Stat Statview 5.0 SAS Institut Inc software. Risk factors for mortality and reintervention were studied by univariate analysis (with contingency coefficient (CC)), using the two-tailed paired Student’s t test and U of Mann–Whitney for the continuous variables and the *χ*2 test for nominal variables, and by multivariate analysis using a logistic regression model (with odds ratio (OR)). The actuarial method was used to determine event-free survival curves. The level of statistical significance was set at a *p* value of less than 0.05.

## Results

### Early mortality and functional status

There were five (6 %) early deaths. Two patients with low cardiac output died on the first postoperative day. A third patient could have been converted to Norwood procedure but died on the first postoperative day because the parents refused the intervention. The last two patients died at 44 and 56 postoperative days due to respectively a severe tricuspid regurgitation with a restrictive mitral profile, and a low cardiac output. These five patients were critically ill preoperatively with severe ventricular dysfunction (two of them needed mechanical ventilation and inotropic support) among whom three needed a long cardiopulmonary bypass (147,161 et 190 min). Four of them required delayed sternal closure.

In fact, three of them were not eligible for a biventricular repair with a diameter of the aortic annulus which was less than five millimeters.

The 2D-echocardiography coupled with Doppler study showed a postoperative mean and maximal peak aortic valve gradient of respectively 24 mmHg (range 10 to 56 mmHg) and 37 mmHg (range 12 to 88 mmHg), significantly lower than before surgery (*p*‹0.0001). There were 58 % of AR grade 1-2/4 and one severe AR 3/4. Seven patients had a SF < 30 % (median 37,5 %, range 21 to 56 %), which was significantly lower than before surgery (7/77 (9 %) vs 45 %, *p* < 0.05).

### Late mortality and reinterventions

There were six late deaths, with a global mortality of 13 % (64 % were neonates). Four of these late deaths occurred early after a second intervention: during Ross intervention in 1, on the first postoperative day after a Ross-Konno procedure because of low cardiac output in the second, five days after a Ross procedure associated with mitral valvuloplasty in the third one, and the fourth one after mitral valvotomy due to pulmonary hypertension. Two other patients died because of pulmonary hypertension : one four years after having two aortic valvotomies and a Ross procedure, and the last one less than a year after aortic valvotomy.

**Fig. 1:**
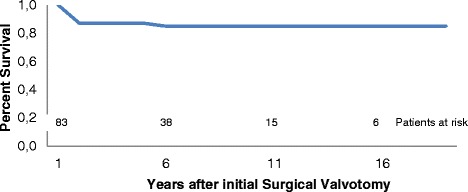
Survival curve after surgical aortic valvotomy

The time-related survival rate was 87 and 85 % at 5 and 15 years, respectively (Fig. 1).

Thirty eight patients had a second intervention (46 %) within a median period of 6.3 months (range 15 days to 14 years), due to:

- Restenosis in 27 patients (13 surgical valvotomies, 6 Ross procedures, 3 Ross-Konno, 5 balloon valvotomies). Concomitant surgery included 7 patches of enlargement of the sinotubular junction, 4 mitral valvotomies and 1 atrial septal defect closure.
- Severe aortic regurgitation (AR grade ≥ 3) in 3 (2 Ross procedures and 1 Ross-Konno).
- Association of aortic stenosis and aortic regurgitation in 7 (three AR grade 2, two AR grade 3 and two AR grade 4) with 6 Ross procedures and 1 surgical valvotomy. Concomitant interventions included 1 patch of enlargement of the sinotubular junction, 2 mitral and 1 tricuspid valvotomy and 1 resection of vegetation.
- Isolated mitral stenosis in 1.

Eleven patients had a third intervention (15.5 %): 3 mitral surgical valvotomy, 1 balloon aortic valvotomy, 1 surgical aortic valvotomy with ventricular septal defect closure and resection of subvalvular aortic stenosis, 1 right ventricle to pulmonary artery Hancock conduit, 1 Ross-Konno procedure, 2 Ross procedures, 1 mechanical aortic valve replacement with enlargement of left pulmonary artery and 1 mechanical mitral valve replacement. There was no death among these patients.

Three patients had a fourth intervention (3.5 %): one left and right coronary artery bypass, one Ross procedure and one right ventricular to pulmonary artery conduit. There was no death.

**Fig. 2:**
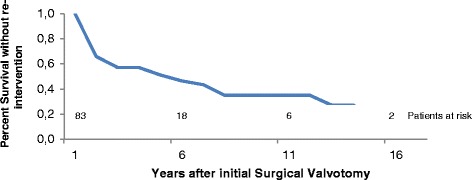
Time-related survival without re-intervention after surgical aortic valvotomy

The time-related survival without reintervention (surgery or balloon valvotomy) was respectively 51, 35 and 18 % at 5, 10 and 15 years (Fig. 2).

**Fig. 3:**
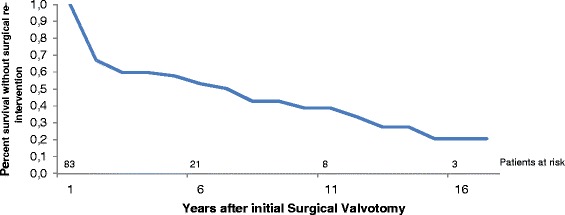
Time-related survival without surgical re-intervention after surgical aortic valvotomy

The time-related survival without surgical reintervention was respectively 58, 39 and 21 % at 5, 10 and 15 years (Fig. 3).

**Fig. 4:**
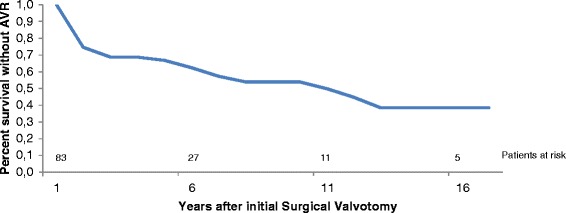
Time-related survival without AVR after surgical aortic valvotomy

The time-related survival without aortic valve replacement (AVR) was respectively 67, 54 and 39 % at 5, 10 and 15 years (Ross or mechanical aortic valve replacement) (Fig. 4).

There is no significant difference in survival, survival without re-intervention and survival without AVR/Ross between before and after 2008.

### Risk factors for global mortality and reintervention

The risk factors studied were: aortic annulus diameter left ventricular dysfunction, SF, inotropic drugs, mechanical ventilation, neonatal period, weight, LVEDD, endocardial fibroelastosis, delayed sternal closure because of the higher risk of mediastinitis,, associated cardiac malformations and prenatal diagnosis.

The bicuspid valve was not studied as a risk factor because of its too high prevalence (84 % of infants had a bicuspid valve). Ventricular dysfunction (*p* = 0.04, CC = 0,2), delayed sternal closure (*p* = 0.007; OR = 21), endocardial fibroelastosis (*p* = 0.02, CC = 0,3), low z-score of the aortic annulus (*p* = 0.04, OR = 0,7) and prenatal diagnosis (*p* = 0,01; OR = 20) were found predictors of global mortality.

**Fig. 5:**
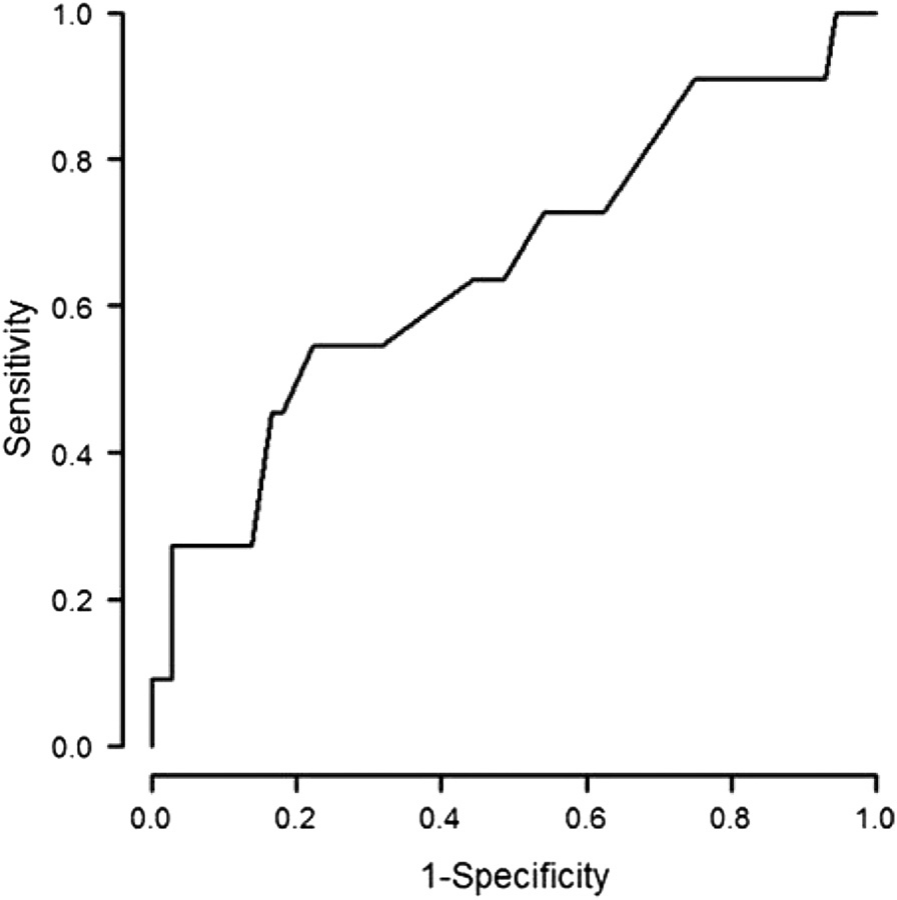
ROC curve of z-score of aortic annulus as risk factor of mortality

The value of z-score of the aortic annulus for which the risk of mortality becomes prohibitive is −3 (specificity: 0,88 and sensitivity: 0,55) with the aera under the curve =0,66 (Fig. 5).

As regards prenatal diagnosis, the result must be interpreted with caution. The percentage of this latter was low (only 17 %) because it is an historic series and only the most severe forms were detected, what explains that the prenatal diagnosis was found predictor of global mortality.

Ventricular dysfunction (*p* = 0.01; OR = 4) and endocardial fibroelastosis (*p* = 0.04; OR = 0,3) were found predictors of re-intervention.

### Long-term functional status

At the latest echocardiographic examination for the 50 patients still having their native aortic valve (69 % of survivors), aortic regurgitation was null in 10, grade 1 in 18, grade 2 in 18, grade 3 in 4. The mean and maximal peak aortic valve gradients were respectively 21 and 41 mmHg.

## Comment

The most appropriate management of critical aortic stenosis in neonates and young infants remains controversial. Since the advent of transcatheter balloon dilatation in 1983 [11], many teams now use exclusively this technique in this group of patients, to avoid the myocardial stress induced by open heart surgery in these often critically ill babies. Only a few studies are focusing only on this age group, but most insist on the high mortality rate in infants, whatever the technique used. Only few recent comparative studies have concentrated on the comparison of these two techniques in neonates [8, 12–14].

In our center, we prefer surgical treatment because of the expertise acquired in that area, and of the higher risk of severe aortic regurgitation with balloon valvotomy. Surgical valvotomy is safe with low mortality and preserves the native aortic valve as long as possible.

Recent studies about infants less than three months of age show a variable rate of early (range from 0 to 19 %) and late mortality with an actuarial survival between 73 and 91 % at 10 years, a freedom from reintervention between 41 and 78 % at 10 years, and freedom from aortic valve replacement (mentioned in only 2 of these studies) between 79 and 83 % at 10 years [4, 8, 15–18].

Siddiqi and al. shows that, after a period where the treatment of choice for valvular aortic stenosis in neonates and infants was balloon dilatation in most centers, the tendency is now shifting in favor of surgical valvotomy, because of the much higher rate of reintervention in patients undergoing balloon valvuloplasty than in surgically treated patients [14].

Our results compare favorably to others for early mortality (6 %) but our rate of reinterventions is higher (freedom from reintervention 35 % at 10 years) with a median delay of 6.3 months, as well as our aortic valve replacement rate (freedom from AVR 54 % at 10 years with a median delay of 1,2 years).

This difference of results can be partially explained by a relatively high rate of severe associated cardiac malformations, notably five multilevel left heart obstruction, one mitral stenosis and one HLHS, more severe hypoplasia of the aortic annulus (median z-score = −1.9) and a majority of bicuspid or monocuspid valves with only 7 % of tricuspid valves.

They seemed to have improved since 2008 with a percentage of re-intervention s and aortic valve replacements decreased by 50 % reaching respectively 28 et 21 %. However, we have finally not found a significant difference in survival, survival without re-intervention and survival without AVR/Ross.

We found that ventricular dysfunction and hypoplastic aortic annulus are predictors of global mortality, along with endocardial fibroelastosis and delayed sternal closure. These results are consistent with those of other studies [8, 14, 16, 18].

The value of z-score of the aortic annulus for which the risk of mortality becomes prohibitive is −3. This amounts to an aortic annulus of 5 mm, which is corresponding with our attitude of surgical management on two ventricles.

This can be illustrated by six patients of our study who had a biventricular repair while the diameter of the aortic annulus was lower than 5 mm; half of them died early.

We prefer to consider the ultrasound measure of the aortic annulus at 5 mm in order to decide a biventricular repair. In fact, according to the method of calculation of the z-score, the value of the latter can change a lot (Detroit, Boston Children's Hospital data,…).

We showed that left ventricular dysfunction and endocardial fibroelastosis were also associated with an increased risk of re-intervention, probably because these anomalies lead to underestimated residual aortic gradient after the aortic valvotomy. This is consistent with the study of Hawkins and al. [16].

A small aortic annulus has been considered by some to be a independent risk factor for earlyreintervention [15, 17], but this was not the case in our study.

We did not find that the neonatal period was a risk factor of global mortality but for others, survival in this category of age is deemed to be inferior to that of infants over one month of life [19, 20]. Unfortunately, this risk factor is poorly studied in the literature. Contrarily to our study, the neonatal period was found as a risk factor of re-intervention in the studies where the balloon dilatation was used, because the priority is then to provide sufficient relief of the ventricular outflow obstruction to allow ventricular recovery while avoiding severe AR [7, 14, 21, 22].

The bicuspid aortic valve was not studied as a risk factor in our study because of its too high prevalence. However, previous reports showed that bicuspid aortic valve was associated with an increased risk of re-intervention [15, 23]. If the surgeon can reconstruct three cusps without producing significant aortic regurgitation, the need for any re-intervention for recurrent stenosis is less than in patients with bicuspid valves [23]. In the study of Hraska and al., freedom from reintervention at 15 years was 100 and 30 % respectively in the patients with tricuspid and monocuspid/bicuspid valves [15].

As in other studies, the main cause for reintervention in our patients group was aortic restenosis. Despite the fact that late mortality was mainly related to reinterventions, several procedures can be performed in the same patient without impairing heart function. Another notable cause of early and late mortality, still not fully understood, is pulmonary hypertension. Endocardial fibroelastosis is common in neonates with critical aortic stenosis. These myocardial changes could explained an increased left ventricular end diastolic pressure and a persistent pulmonary hypertension several years after aortic valvotomy without recurrent left ventricular outflow tract obstruction [24]. Pulmonary hypertension is a well known consequence of restrictive cardiomyopathy [25] and isolated endocardial fibroelastosis [26]. Perhaps, we should consider additional surgical approaches such as left ventricular endocardial resection in order to limit the ventricular remodeling and the development of diastolic left ventricular dysfunction.

### Balloon valvotomy

Many centers use balloon dilatation in order to treat critical aortic stenosis in infants. These centers display good results with low mortality and low rate of severe aortic regurgitation [5–7]. In the study of Mc Elhinney and al. about 113 infants under 2 months of age, freedom from surgical reintervention and aortic valve replacement was respectively 64 and 76 % at 10 years and early mortality was 4 % between 1994 and 2002 [7].

### Ross Procedure in Neonates and Infants

Ross procedure is an attractive surgical choice because of the advantages of optimal size match between the pulmonary autograft and the smaller aortic annulus; these patients’ pulmonary valves may be better suited to systemic pressures than the valves of older patients. However, the disadvantages include placing two valves at risk for a single valve disease and the risk of late dilatation of the autograft, which may result in aortic insufficiency [27–29].

Aortic valve replacement in neonates and young infants is associated with substantial mortality, ranking among the highest risk of all cardiac procedures in these age groups [30].

However, in the study of Shinkawa and al. concerning Ross procedure in 31 neonates and infants, the intermediate-term results are acceptable with actuarial survival rate at 10 years of 76.7 % and freedom from reoperation of 50.6 % at 10 years [31].

### Two-stage biventricular rehabilitation for severe left ventricular dysfunction

Ventricular dysfunction is common in critical aortic stenosis and is associated with higher mortality regardless of the type of management (surgical or balloon valvotomy). Some centers have reported a two-stage biventricular rehabilitation (surgical or hybrid approaches) in order to improve recovery of left ventricular dimensions and contractility, and also improve right ventricular hemodynamics during the temporary right ventricular-dependent systemic circulation. However, these are still experimental studies with low numbers of patients [32–34].

## Study limitations

This is a retrospective single-center study with loss of data in the medical records.

## Conclusion

The experience, in our center, on the management of critical aortic stenosis, shows a low early and late mortality (actuarial survival rate at 15 years: 85 %), but the aortic valvotomy is a palliative procedure and we see unfortunately a high rate of reintervention among which the aortic valve replacement. These results suggest to reconsider the use of aortic balloon valvotomy, and particularly for the neonates with a low cardiac output in order to avoid the myocardial stress and the neurological injury due to the cardiopulmonary bypass.
